# Echocardiographic assessment of left ventricular volumes: a comparison of different methods in athletes

**DOI:** 10.1007/s00392-024-02504-4

**Published:** 2024-08-05

**Authors:** Joscha Kandels, Verena Denk, Maria Weinkouff Pedersen, Kristian Hay Kragholm, Peter Søgaard, Bhupendar Tayal, Robert Percy Marshall, Timm Denecke, Filip Lyng Lindgren, Andreas Hagendorff, Stephan Stöbe

**Affiliations:** 1 Department of Cardiology, Leipzig University Hospital, Liebigstr. 20, 04103 Leipzig, Germany; 2 Department of Cardiology, Aalborg University Hospital, Hobrovej 18-22, 9000 Aalborg, Denmark; 3 Department of Cardiology, Aarhus University Hospital, Palle Juul-Jensens Boulevard 99, 8200 Aarhus N, Denmark; 4 Unit of Clinical Biostatistics and Epidemiology, Aalborg University Hospital, Hobrovej 18-22, 9000 Aalborg, Denmark; 5 Cleveland Medical Center, Harrington Heart and Vascular Institute, University Hospitals, Cleveland, OH USA; 6 RasenBallsport Leipzig GmbH, Cottaweg 3, 04177 Leipzig, Germany; 7 Department of Orthopedic and Trauma Surgery, Martin-Luther-University Halle-Wittenberg, Ernst-Grube-Straße 40, 06120 Halle, Germany; 8 Department of Diagnostic and Interventional Radiology, University Hospital Leipzig, 04103 Leipzig, Germany; 9 Department of Cardiology, North Denmark Regional Hospital, Bispensgade 37, 9800 Hjørring, Denmark

**Keywords:** Transthoracic 2D and 3D echocardiography, Doppler echocardiography, Left ventricular volumes, Cardiac magnetic resonance imaging, Athletes

## Abstract

**Background:**

Cardiac magnetic resonance imaging (cMRI) is considered the gold standard for the assessment of left ventricular (LV) systolic function. However, discrepancies have been reported in the literature between LV volumes assessed by transthoracic echocardiography (TTE) and cMRI. The objective of this study was to analyze the differences in LV volumes between different echocardiographic techniques and cMRI.

**Methods and results:**

In 64 male athletes (21.1 ± 4.9 years), LV volumes were measured by TTE using the following methods: Doppler echocardiography, anatomical M-Mode, biplane/triplane planimetry and 3D volumetry. In addition, LV end-diastolic (LVEDV), end-systolic (LVESV), and stroke volumes (LVSV) were assessed in 11 athletes by both TTE and cMRI. There was no significant difference between LVEDV and LVESV determined by biplane/triplane planimetry and 3D volumetry. LVEDV and LVESV measured by M-Mode were significantly lower compared to 3D volumetry. LVSV determined by Doppler with 3D planimetry of LV outflow tract was significantly higher than 2D planimetry and 3D volumetry, whereas none of the planimetric or volumetric methods for determining LVSV differed significantly. There were no significant differences for LVEDV, LVESV, LVSV and LVEF between cMRI and TTE determined by biplane planimetry in the subgroup of 11 athletes.

**Conclusion:**

The choice of echocardiographic method used has an impact on LVSV in athletes, so the LVSV should always be checked for plausibility. The same echocardiographic method should be used to assess LVSV at follow-ups to ensure good comparability. The data suggest that biplane LV planimetry by TTE is not inferior to cMRI.

## Introduction

Quantitative echocardiography is becoming increasingly prevalent in clinical studies. Quantitative parameters such as left ventricular (LV) ejection fraction (LVEF), regurgitant volumes and regurgitation fraction are used to define therapeutic decision criteria, for instance in patients with heart failure and valvular regurgitations [1, 2]. From both a medical and an ethical point of view, therapeutic decisions should be based on hemodynamically plausible echocardiographic data [3, 4]. However, data from the large prospective transcatheter edge-to-edge repair (TEER) studies COAPT and MITRA-FR [5, 6] demonstrate that, despite the hemodynamic echocardiographic inconsistencies observed in these studies, the results have led to treatment recommendations in current guidelines [7].

The discrepancies observed in echocardiographic findings may be attributed to improper quantitative assessment of cardiac volumes. The frequent inconsistencies observed in the assessment of valvular heart disease (VHD), particularly in case of valvular regurgitations, represent a major weakness of the so-called integrative approach in echocardiography [8]. Consequently, cardiac magnetic resonance imaging (cMRI) is currently considered the preferred method in selected patients according to the 2017 ASE guidelines [2]. In contrast, transthoracic echocardiography (TTE) can use the same approaches to determine cardiac volume as cMRI [9]. The general problem of determining cardiac volumes by 2D and 3D TTE planimetry or volumetry is also evident when considering the normal values reported in the literature [9]. The mean values of LV stroke volume (LVSV) are occasionally below [9], in the range of [10] or slightly above [10–12] the cardiac index (CI) threshold of 2.2 l/min/m^2^ [13–16]. However, normal values of LVSV as determined by 3D TTE are usually within hemodynamically plausible ranges [10, 17, 18]. In comparison to studies using echocardiographic planimetry or volumetry, Doppler echocardiographic studies have documented LVSV, cardiac output (CO) and CI values within normal ranges comparable to those obtained by other techniques, such as thermodilution and the Fick method [19, 20]. In addition, the lower cardiac volumes documented in the literature for 2D and 3D TTE compared to cMRI suggest that 2D TTE planimetry and 3D TTE volumetry may underestimate cardiac volumes due to methodological errors or poor visualization due to lower spatial resolution [21–24].

In view of the known discrepancies and inconsistencies in the echocardiographic assessment of LV volumes [3–6, 9, 10], the objective of this study was to analyze the differences in LV volume determination using different echocardiographic methods in healthy athletes.

## Methods

A total of 64 healthy athletes (soccer players: *n* = 58, handball players: *n* = 4, ballet dancers: *n* = 2) underwent TTE at the University Hospital of Leipzig between January 2020 and February 2022 using Vivid e95 ultrasound systems with M5-S and MV phased-array probes (GE Healthcare Vingmed Ultrasound AS, Horten, Norway).

Data analysis was performed using the EchoPac software (version 204 and 206, GE Healthcare Vingmed Ultrasound AS, Horten, Norway). This involved length and area measurements in DICOM images for M-Mode and 2D image analysis, as well as 4D-LVQ for 3D volume analysis. All participants showed no evidence of structural heart disease. Participants with a VHD grade of more than trace were excluded. The main inclusion criterion for the present study was a sufficient image quality allowing the accurate and verifiable measurement of LV end-diastolic and end-systolic volumes (LVEDV, LVESV) and LVSV with all modalities. Basic parameters, including body height and body weight, were documented to calculate body surface area (BSA) and body mass index (BMI). Resting heart rate (HR), electrocardiogram (ECG) and blood pressure by manual sphygmomanometry (systolic blood pressure—BP_sys_ and diastolic blood pressure—BP_dia_) were recorded within 5 min before TTE. To compare stroke volumes in healthy subjects without VHD, it is essential to ensure that total SV, forward SV and effective SV are identical.

LVSV was measured in all participants using the following approaches: LVSV by forward stroke volume with Doppler echocardiography, by M-Mode analysis (LVSV_M-Mode_) using the Teichholz formula *V* = 7*D^3^/(2.4 + *D*), whereas *D* is the inner LV diameter, by manual Simpson biplane 2D planimetry (LVSV_biplane_), by simultaneous triplane planimetry (LVSV_triplane_) as well as by 3D volumetry with manual corrections (LVSV_3D-volumetry_). With respect to the two approaches for the assessment of the cross-sectional area (CSA) of the LV outflow tract (LVOT) by 2D and 3D echocardiography 6 different methods for determining the effective LVSV were used.

A subgroup of 11 athletes underwent cMRI (Philips Healthcare Achieva 3.0 T system, Amsterdam, The Netherlands) at the same day of TTE. All athletes had a clinical indication for cMRI for suspected viral myocarditis, which was subsequently ruled out by Lake-Louise criteria. Data analysis was performed using IntelliSpace PACS software (version 4.4, Philips Healthcare, Amsterdam, The Netherlands).

### Assessment of LVEDV, LVESV and LVSV by anatomical M-mode

**Fig. 1:**
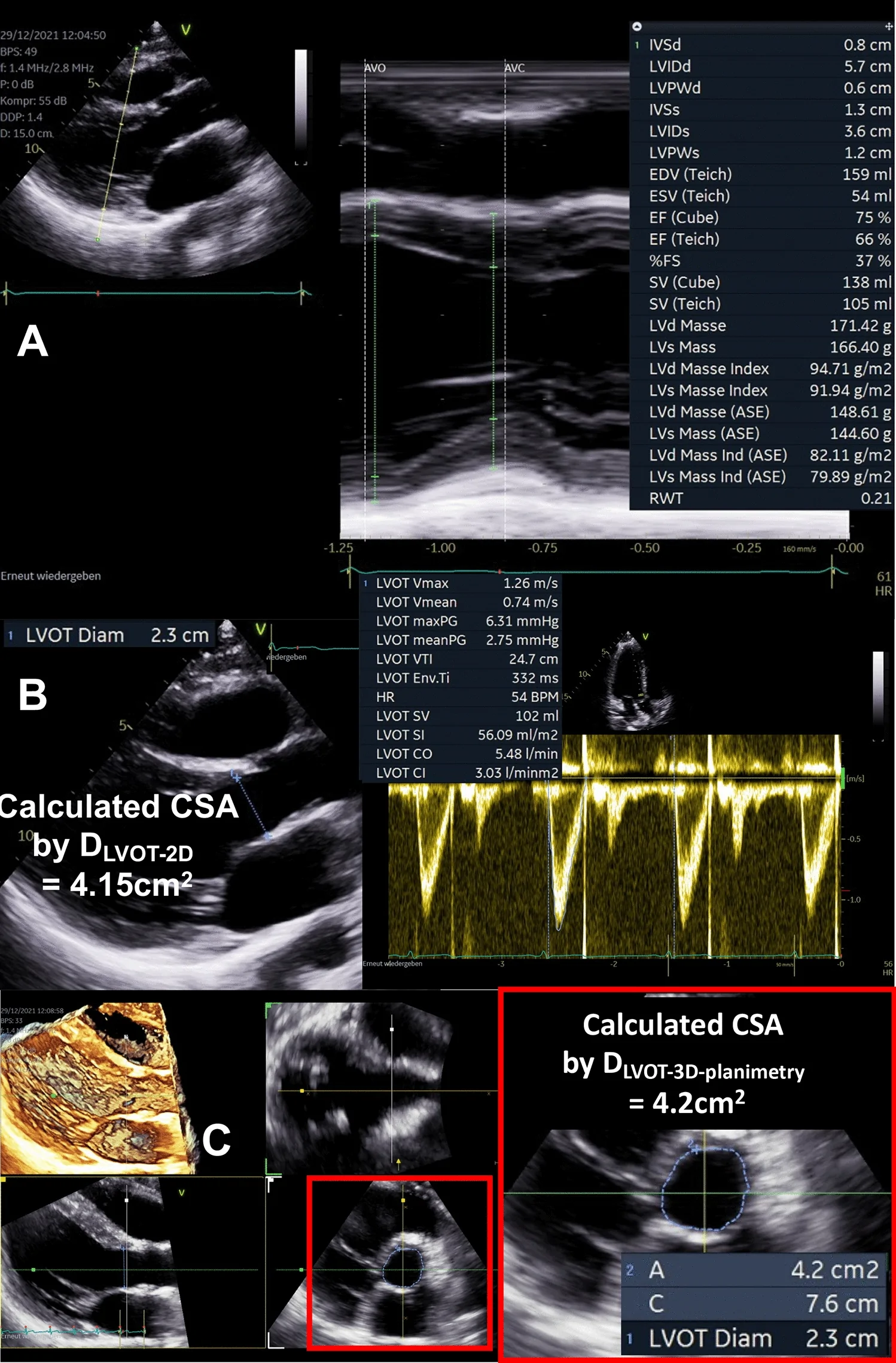
Left ventricular (LV) stroke volume (LVSV) assessment by anatomical M-Mode using Teichholz equation (**A**) and LVSV assessment by Doppler echocardiography using 2D measurements of LVOT (left ventricular outflow tract) diameter (D_LVOT-2D_) in a standardized 2D parasternal long axis view by 2D echocardiography and LVOT planimetry of cross-sectional area (CSA_LVOT-3D-planimetry_) of the LVOT by 3D echocardiography and the velocity time integral (VTI) of LVOT velocities using pulsed wave (pw) Doppler using the apical long axis view. In **B** and **C**, the measurements of LVSV by 2D and 3D echocardiography are shown using the following equations: LVSV_PWDE_ = CSA_LVOT-2D-calculated_ × VTI_LVOT_ = *π* × (*D*_LVOT-2D_/2)^2^ × VTI_LVOT_ and LVSV_PWDE_ = CSA_LVOT-3D-planimetry_ × VTI_LVOT_

Measurements were performed in a standardized parasternal long axis view (pLAX) using anatomical M-Mode for calculation of LVEDV_M-Mode_, LVESV_M-Mode_ and LVSV_M-Mode_ illustrated in Fig. 1. The proper sectional plane was controlled by previous biplane parasternal scanning to exclude LV secant sectional planes. Measurements included LV internal cavity diameter, LV interventricular septum thickness and LV posterior wall thickness during diastole (LVIDd, IVSd, LVPWd) and systole (LVIDs, IVSs, LVPWs). LV remodeling index (LVRI) was obtained by calculating the ratio of LV mass (LVM) to LVEDV [25–27].

### LVSV assessment by Doppler echocardiography

LVSV was calculated by multiplying the CSA of the LVOT by the velocity time integral (VTI) of the blood flow velocity in the LVOT determined by pulsed wave Doppler echocardiography (PWDE). The CSA of the LVOT was calculated by the antero-posterior diameter (D_LVOT_2D) of the LVOT determined in the pLAX during mid systole by 2D TTE (CSA_LVOT-2D-calculated_) or by planimetry of the LVOT area in the adjusted sectional plane within 3D data sets (CSA_LVOT-3D-planimetry_) (Fig. 1). In addition, the geometric opening area (GOA) of the aortic valve (AV) was determined by planimetry in the adjusted sectional planes of 3D data sets to compare CSA of the respective outflow tracts and the GOAs of the AV.

The following equations were used:

$${\mathrm{LVSV}}_{{{\mathrm{PWDE}}}} = {\text{ CSA}}_{{{\mathrm{LVOT}} - {\mathrm{2D}} - {\mathrm{calculated}}}} \times {\text{ VTI}}_{{{\mathrm{LVOT}}}} = \pi \times \, (D_{{{\mathrm{LVOT}}}} {2}D/{2})^{{2}} \times {\mathrm{VTI}}_{{{\mathrm{LVOT}}}}$$

$${\mathrm{LVSV}}_{{{\mathrm{PWDE}}}} = {\mathrm{CSA}}_{{{\mathrm{LVOT}} - {\mathrm{3D}} - {\mathrm{planimetry}}}} \times {\mathrm{VTI}}_{{{\mathrm{LVOT}}}}$$

CSA_LVOT-3D-planimetry_ was compared to the GOA of the aortic valve determined by planimetry of the corresponding orifice areas in the adjusted sectional planes within 3D data sets.

### Cardiac volume assessment by 2D planimetry and by 3D volumetry

**Fig. 2:**
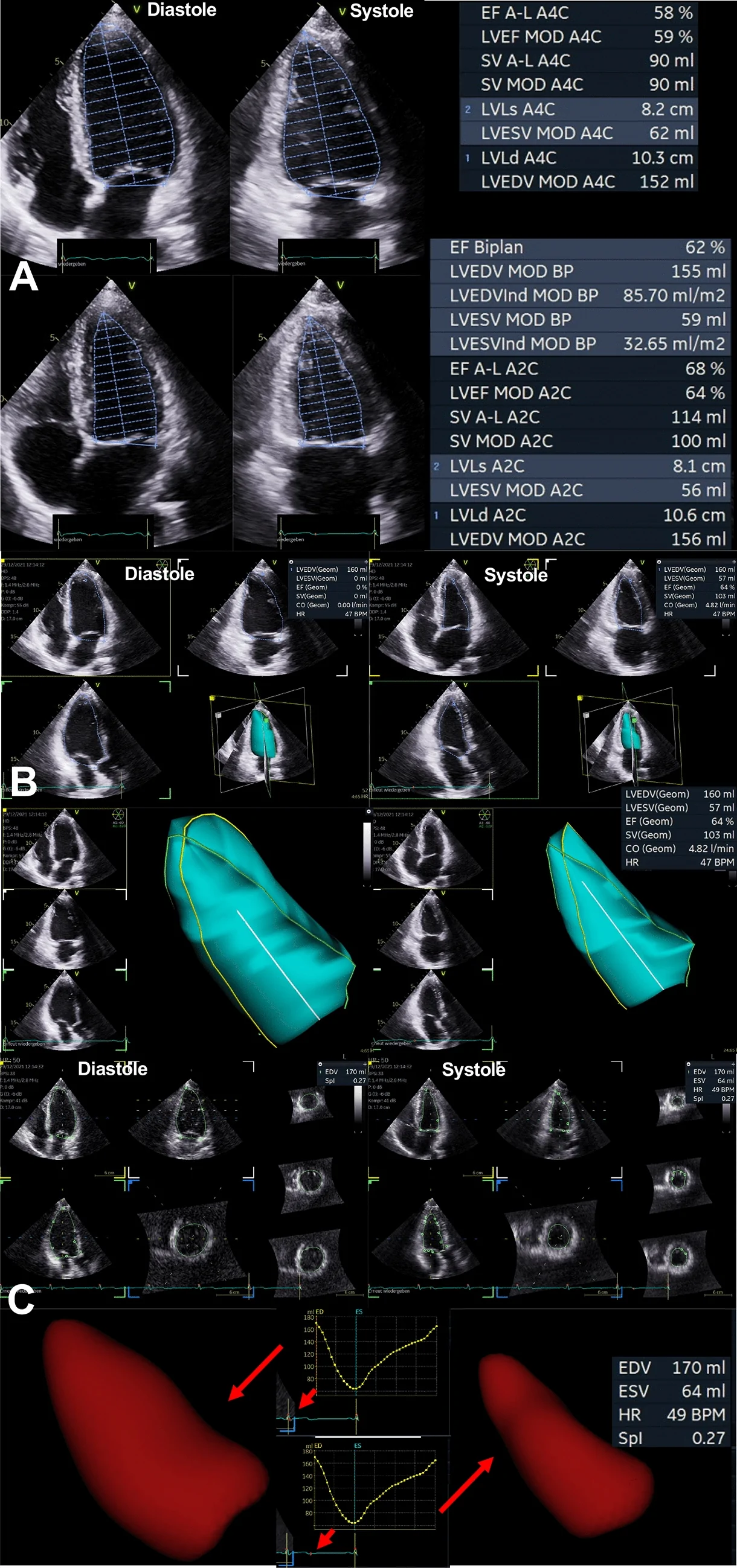
Biplane LV planimetry using Simpson’s method (2Ch and 4Ch) in **A**. The calculated values of LV end-diastolic and end-systolic volume as well as stroke volume (LVEDV, LVESV, LVSV) are presented. Simultaneous triplane LVSV assessment in **B** and of 3D LVSV assessment in **C**. The calculated values of LV end-diastolic and end-systolic volume as well as stroke volume (LVEDV, LVESV, and LVSV) are presented for each method

LVEDV, LVESV, LVSV and LVEF were assessed by biplane planimetry (LVEDV_biplane_, LVESV_biplane_, LVSV_biplane_) in the apical two- and four-chamber view (2ChV, 4ChV) using the modified Simpson’s rule, by simultaneous triplane planimetry (LVEDV_triplane_, LVESV_triplane_, LVSV_triplane_) and by 3D volumetry (LVEDV_3D-volumetry_, LVESV_3D-volumetry_, LVSV_3D-volumetry_) using 3D data sets. In all methods, calculations were based on manually traced endocardial contours at end diastole and end systole. Endocardial contours were labeled at the border between non-compacted and compacted myocardium. LVSV assessment by bi- and triplane 2D planimetry and 3D volumetry is illustrated in Fig. 2.

### Cardiac volume assessment by cardiac magnetic resonance tomography

**Fig. 3:**
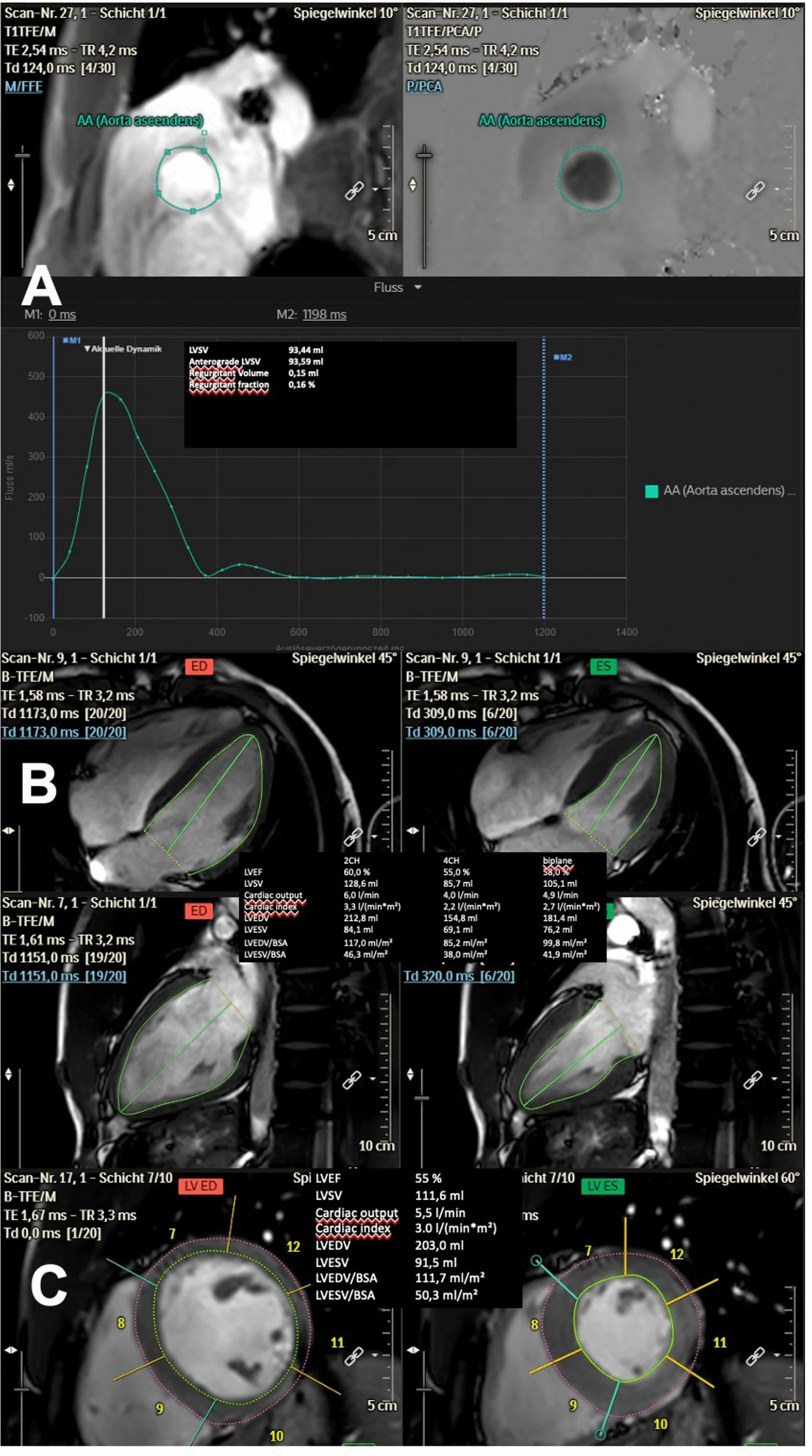
Flow measurements (phase contrast sequences) and left ventricular planimetries of left ventricular (LV) stroke volume (LVSV) in cardiac magnetic resonance imaging (cMRI). The corresponding flow measurement including the values are presented depicted in **A**. The biplane LV planimetry of the 4- and 2-chamber view (left—end diastole; right—end systole) is presented in **B**, the respective values, including indexed volume parameters are given (*LVEF* LV ejection fraction, *LVEDV* LV end-diastolic volume; *LVESV* LV end-systolic volume). The respective measurements of the short axis planimetry are presented in **C**

LVSV was estimated by the calculation of the volume flow Q through the LVOT using retrospectively gated phase contrast cMRI methods that are sensitized to through plane velocities by obtaining a thin, cross-sectional image obtained in transversal orientation at the aortic annulus. This CSA is covered by a set of pixels. The LVSV is calculated by summing all velocity pixels that cover the CSA during systole by the equation LVSV = CSA × Q. In addition, LVEDV, LVESV and LVSV were determined by cMRI using steady-state free precession technique. Biplane LV planimetry in the 2- and 4ChV as well as by short axis-based planimetry with end-diastolic and end-systolic contour delineation were performed to compare methodological approaches between cMRI and TTE. LVSV assessment by cMRI is illustrated in Fig. 3. For both echocardiography and cMRI, image acquisition was performed during breath-hold maneuvers. In particular, 3D echocardiography for stitched LV volume assessment required a breath-hold maneuver for six heartbeats, cMRI cineloop acquisition for up to 12 heartbeats.

### Statistical analysis

All statistical analyses were performed using SPSS Statistics (version 29.0.2.0 (20), IBM, Armonk, New York). Permanent variables were expressed as mean value ± standard deviation (SD) and were compared between the different methods using Student’s *t* test (two-tailed). Data comparisons between more than two groups were performed by one-way analysis of variance (ANOVA) followed by Bonferroni post hoc analyses. A *p* value < 0.05 was considered to indicate statistical significance. The kappa coefficient (*κ*) was used to assess intra- and interobserver variability for TTE and cMRI measurements.

## Results

Baseline data and basic echocardiographic parameters are given in Table 1. Cardiac geometry is represented by mild to moderate LV dilatation and mild LV hypertrophy characterizing mild forms of athlete's hearts [28–31]. An athlete’s heart is usually defined as a heart volume ≥ 12 ml/kg body weight in women and ≥ 13 ml/kg body weight in men with maximum values of 19 ml/kg and 20 ml/kg, respectively [32].

**Table 1:** Baseline characteristics and basic echocardiographic parameters

Parameters	Mean value ± SD
Age (years)	21.1 ± 4.9
Sex (male)	64 (100%)
Weight (kg)	78.3 ± 9.8
Height (cm)	182.6 ± 7.1
BMI (kg/m^2^)	23.4 ± 1.8
BSA (m^2^/kg)	2.0 ± 0.2
HR (bpm)	58.7 ± 8.1
BPs (mmHg)	129.4 ± 9.4
BPd (mmHg)	72.3 ± 8.6
LVEF (%)	61.3 ± 6.6
LVOT VTI (m/s)	25.41 ± 3.28
LVEDD (mm)	56.5 ± 3.7
IVSD (mm)	8.4 ± 1.2
LVPWD (mm)	7.7 ± 1.1
E/E'	6.4 ± 1.6
E/A	2.1 ± 0.8
LVMI (g/m^2^)	100.5 ± 17.5
RWT	0.28 ± 0.04
LVRI (g/ml)	1.3 ± 0.2
sPAP (mmHg)	28.2 ± 3.8

*BMI* Body mass index, *BSA* Body surface area, *HR* Heart rate, *BPs* blood pressure systolic, *BPd* blood pressure diastolic, *LVEF* left ventricular ejection fraction, *LVOT* *VTI* left ventricular outflow tract velocity time integral, *LVEDD* left ventricular end-diastolic diameter, *IVSD* interventricular septal diameter, *LVPWD* left ventricular posterior wall diameter, *LVMI* left ventricular mass index, *RWT* relative wall thickness, *LVRI* left ventricular remodeling index, *sPAP* systolic pulmonary arterial pressure

There was no difference between CSA measurements for Doppler assessment of forward LVSV (*p* = 0.353): CSA_LVOT-2D-calculated_ (4.3 ± 0.8 cm^2^) compared to CSA _LVOT-3D-planimetry_ (4.5 ± 0.8 cm^2^). The GOA_AV_ assessed by planimetry (4.0 ± 0.7 cm^2^) was smaller compared to the calculated CSA_LVOT-2D-calculated_ (*p* = 0.007) and CSA _LVOT-3D-planimetry_ (p < 0.001). LVRI did not differ between athletes with African roots (*n* = 18, 28%) and other athletes (1.3 ± 0.3 g/ml vs. 1.2 ± 0.2 g/ml; *p* = 0.123).

The results of LVEDV and LVESV assessment by 2D planimetry (biplane and triplane method) and 3D volumetry are presented in Table 2. Although the highest values of LVEDV and LVESV were determined by 3D volumetry, there was no difference between LVEDV (*p* = 0.160) and LVESV (*p* = 0.060) determined by 2D planimetry and 3D volumetry.

**Table 2:** Left ventricular end-diastolic and end-systolic volumes determined by different echocardiographic methods in 64 athletes

	M-Mode	Biplane	Triplane	3D volumetry
LVEDV Mean value ± SD (ml)	158.4 ± 23.5 ml *	165.5 ± 27.8	164.8 ± 27.1	173.2 ± 26.8
LVESV Mean value ± SD (ml)	51.8 ± 12.8 ml *,†,‡	64.2 ± 16.2	64.6 ± 17.6	70.5 ± 16.6

*LVEDV* left ventricular end-diastolic volume, *LVESV* left ventricular end-systolic volume. For LVEDV: *M-Mode compared to 3D volumetry (*p* = 0.010). For LVESV: * M-Mode compared to Biplane (*p* < 0.001); † M-Mode compared to Triplane (*p* < 0.001); ‡ M-Mode compared to 3D volumetry (*p* < 0.001)

In contrast, LVEDV by M-Mode was lower compared to LVEDV_3D-volumetry_ (158.4 ± 23.5 ml vs. 173.2 ± 26.8 ml; *p* = 0.010). Further, LVESV determined by M-Mode was lower compared to LVESV_biplane_ (51.8 ± 12.8 ml vs. 64.2 ± 16.2 ml; *p* < 0.001), LVESV_triplane_ (51.8 ± 12.8 ml vs. 64.6 ± 17.6 ml; *p* < 0.001) and LVESV_3D-volumetry_ (51.8 ± 12.8 ml vs. 70.5 ± 16.6 ml; *p* < 0.001).

The results of LVSV assessment by the different methods are presented in Table 3. The highest discrepancy between the mean LV stroke volume values obtained by all echocardiographic methods was 13.1 ml. The highest LVSV was determined by Doppler echocardiography (113.3 ± 23.1 ml), the lowest by 2D triplane planimetry (100.2 ± 18.8 ml). None of the planimetric or volumetric methods for determining LVSV differed significantly. LVSV determined by VTI_LVOT_ and the CSA _LVOT-3D-planimetry_ was higher compared to biplane (*p* = 0.012), triplane planimetry (*p* = 0.004) and 3D volumetry (*p* = 0.042), but not compared to LVSV determined by VTI_LVOT_ and the CSA_LVOT-2D-calculated_ (*p* = 0.370) and LVSV determined by M-Mode (*p* = 0.060).

**Table 3:** Determination of left ventricular stroke volume by different echocardiographic methods in 64 athletes

Methods of LVSV assessment	*Mean value* ± *SD (ml)*
LVSV determined by anatomical M-Mode	106.5 ± 17.0
LVSV determined by VTI_LVOT_ and CSA_LVOT-2D-calculated_	109.9 ± 20.6
LVSV determined by VTI_LVOT_ and CSA_LVOT-3D-planimetry_	113.3 ± 23.1
LVSV determined by biplane 2D Simpson’s method	101.3 ± 20.3
LVSV determined by triplane planimetry	100.2 ± 18.8
LVSV determined by 3D LV volumetry (man.)	102.6 ± 20.3

*LVSV* left ventricular stroke volume, *VTI* velocity time integral, *D* diameter, *CSA* cross-sectional area, *LAX* apical long axis, *2Ch* two chamber, *4Ch* four chamber

### Comparison of left ventricular volumes assessed by cMRI and TTE

LVEDV (191.3 ± 29.5 ml vs. 197.1 ± 24.5 ml; *p* = 0.620), LVESV (68.8 ± 17.0 ml vs. 78.5 ± 16.8 ml; *p* = 0.196), LVSV (122.5 ± 23.8 ml vs. 118.5 ± 15.7 ml; *p* = 0.654) and LVEF (64.0 ± 7.1 ml vs. 60.3 ± 5.4 ml; *p* = 0.188) did not differ between Simpson’s method by TTE and cMRI. In addition, LV volumes were also assessed in short axis views by cMRI (LVEDV 185.7 ± 23.9 ml, LVESV 77.0 ± 10.0 ml and LVSV 108.7 ± 20.8 ml) without showing any differences between LVSV determined by cMRI using biplane compared to short axis analysis (*p* = 0.225). Cardiac MRI measurements are illustrated in Fig. 3. There was no difference when comparing LVSV assessed by cMRI with all echocardiographic methods (*p* = 0.190) (Table 4).

**Table 4:** Determination of left ventricular stroke volume in 11 athletes who underwent both, echocardiography and cMRI

Methods of LVSV assessment	Mean value ± SD (ml)	*p* value
LVSV determined by cMRI (biplane planimetry)	118.5 ± 15.7	0.190
LVSV determined by cMRI (short axis views)	108.7 ± 20.8	
LVSV determined by Q-Flow by cMRI	111.3 ± 20.4	
LVSV determined by anatomical M-Mode	116.1 ± 19.0	
LVSV determined by VTI_LVOT_ and CSA_LVOT-2D-calculated_	127.8 ± 23.6	
LVSV determined by VTI_LVOT_ and CSA_LVOT-3D-planimetry_	131.7 ± 26.9	
LVSV determined by biplane 2D Simpson’s method	122.5 ± 23.8	
LVSV determined by triplane planimetry	121.3 ± 13.8	
LVSV determined by 3D LV volumetry (man.)	125.2 ± 19.9	

*LVSV* left ventricular stroke volume, *CMR* cardiac magnetic resonance imaging, *VTI* velocity time integral, *D* diameter, *CSA* cross-sectional area, *LAX* apical long axis, *2Ch* two chamber, *4Ch* four chamber

Intra- and interobserver variability showed a high agreement for echocardiographic assessment of LVSV by 2D planimetry (*κ* = 0.87; *z* = 4.66, *p* < 0.001; *κ* = 0.78; *z* = 4.29, *p* < 0.001) and by biplane cMRI (*κ* = 0.86; *z* = 4.57, *p* < 0.001; *κ* = 0.77; *z* = 4.19, *p* < 0.001). Intra- and interobserver variabilities for the remaining conventional echocardiographic measurements consistently showed good agreement as well.

## Discussion

The main findings of the present study are:

1. There was no significant difference between LVEDV and LVESV determined by 2D planimetry (biplane and triplane method) and 3D volumetry. However, LVEDV and LVESV by M-Mode analysis were significantly lower, especially compared to 3D volumetry.
2. Echocardiographic assessment of LVSV by VTI_LOVT_ and CSA_LVOT-3D-planimetry_ resulted in significant higher values compared to 2D planimetry and 3D volumetry.
3. There were no significant differences for LVEDV, LVESV, LVSV and LVEF between cMRI and TTE determined by biplane planimetry.
4. Assessment of CSA_LVOT_ did not significantly differ between 2 and 3D TTE. The GOA_AV_ assessed by planimetry was significantly smaller compared to CSAs of the LVOT.

### Methodological prerequisites in echocardiography to ensure verifiable cardiac volume measurements

The mathematical assumptions underlying M-Mode measurements of LV volume can only be applied when cardiac geometry and function are close to normal [33, 34]. The cardiac geometry in the present cohort represents mild to moderate LV dilatation and mild LV hypertrophy as evidenced by increased LV end-diastolic diameter (LVEDD), LV mass index (LVMI), LVRI, LVEDV and LVSV values characterizing a mild form of an athlete’s heart [25, 28, 30]. However, a LV hypertrophy induced mainly by strength training—known as the Morganroth hypothesis [35, 36]—is still debatable in athlete’s heart [30, 37, 38]. LVRI indicates a slight concentric LV remodeling, which can be explained by a significant proportion of strength training, but also by a relevant proportion of athletes of African origin [31]. However, the relative wall thickness (RWT) in the present cohort was relatively low within the normal ranges underscoring the reason for questioning the Morganroth hypothesis. Therefore, this low normal RWT value might be due to a cohort of athletes characterized by short-term training episodes as a predominant role. M-Mode analysis seems to be suitable for estimation of LV mass, LV volumes and LVRI when LV geometry is close to normal [27]. In cases of abnormal LV geometry, LV mass assessed by 3D echocardiography [25] and by cMRI can probably characterize LV remodeling more accurately, using different formulas for LVRI determination [39]. In any case, for M-Mode measurement, even in healthy subjects and athletes, it is important to standardize the sectional plane of the maximum LV diameter perpendicular to the LV long axis and at the level of the tips of the open mitral valve [11]. The appropriate sectional plane for a correct anatomical M-Mode analysis can be ensured using 3D or biplane scanning techniques [17, 18].

The Doppler assessment of LVSV includes two essential prerequisites for the determination of appropriate values: (1) the ultrasound beam has to be almost parallel to the direction of blood flow. According to the Doppler equations, a 15° angle between the direction of the ultrasound beam and the direction of blood flow results in a 3% reduction in measured velocities, a 30° angle in a 13% reduction; (2) the sample volume of a 5 mm gate should be positioned properly in the region of the CSA_LVOT_. For a better standardization of positioning the sample volume, the apical long axis view seems to be advantageous, as the positioning of the sample volume in relation to the profile view of the aortic valve can only be verified in this view. The sample volume should be positioned in the septal third of the LVOT to minimize errors in ultrasound Doppler angulation and the aortic valve closure signal should be seen in the Doppler spectrum to document the correct position of the sample volume corresponding to the same level as the CSA_LVOT_ measurement [40, 41]. Despite many methodological prerequisites of PWDE, this method provides the highest LVSV values, confirming data from previous studies [19, 20].

2D LV planimetry and 3D volumetry are highly dependent on good image quality and correct delineation of the plane of the mitral annulus and the endocardial contours at the border between non-compacted and compacted myocardium [11]. However, despite considering these methodological aspects, cardiac volumes may be underestimated by 2D planimetry and 3D volumetry compared to CO measurements by Doppler echocardiography [20, 40, 42, 43]. It can be assumed that the main error in endocardial border delineation occurs during end diastole, as LV foreshortening in 2D echocardiography results in lower LVEDV values. In addition, the distances between the myocardial trabeculae can often not be clearly visualized by 2D and 3D echocardiography due to limited spatial resolution. However, in the present study, the highest LVEDV values were obtained by methods with endocardial border delineation. The most used method for LV volume analysis is still the 2D biplane planimetry using the Simpson’s method. To avoid underestimation of LV volumes by the Simpson’s method, standardization of apical 2- and 4ChV should be verified by simultaneous triplane scanning. It is recommended to check the difference between end-diastolic and end-systolic LV long axis length, which should be less than 5 mm in all apical LV views to exclude LV foreshortening [11].

The analysis of LV volumes and LV mass by 3D echocardiography is often limited by lung overlay of the lateral LV wall. The assessment of CSA by 3D echocardiography is not yet established. However, direct planimetry of the CSA_LVOT-3D-planimetry_ seems to be feasible, especially for the LVOT. Nevertheless, standardization of surface rendering in relation to ultrasound settings, e.g., ultrasound frequencies and gain settings, by phantom studies may be necessary to minimize the impact of pixel attenuation and blooming and to ensure a proper delineation of myocardial surfaces [44, 45].

### Differences of cardiac volumes between TTE and cMRI

Most comparisons in the literature between echocardiography and cMRI have shown an underestimation of cardiac volumes by echocardiography or an overestimation by cMRI, respectively [43, 46–51]. Despite the lack of evidence as to which method can correctly determine cardiac volumes when used correctly, cMRI is considered the gold standard [52]. In the present subcohort of healthy athletes with sufficient image quality, no significant differences were found in the measurement of LV volumes and LVEF between TTE and cMRI by biplane planimetry. This suggests that the differences described in the literature are probably due to errors resulting from methodological limitations, e.g., limited spatial resolution, image quality and lack of standardization in 2D TTE. Surprisingly, although cMRI is supposed to be the gold standard, an additional comparison of cMRI measurements of LV volumes (by short axis planimetry in this cohort) showed lower LVEDV and LVSV values compared to biplane LV planimetry of both modalities.

### Proposal for a possible algorithm to assess left ventricular (stroke) volume

**Fig. 4:**
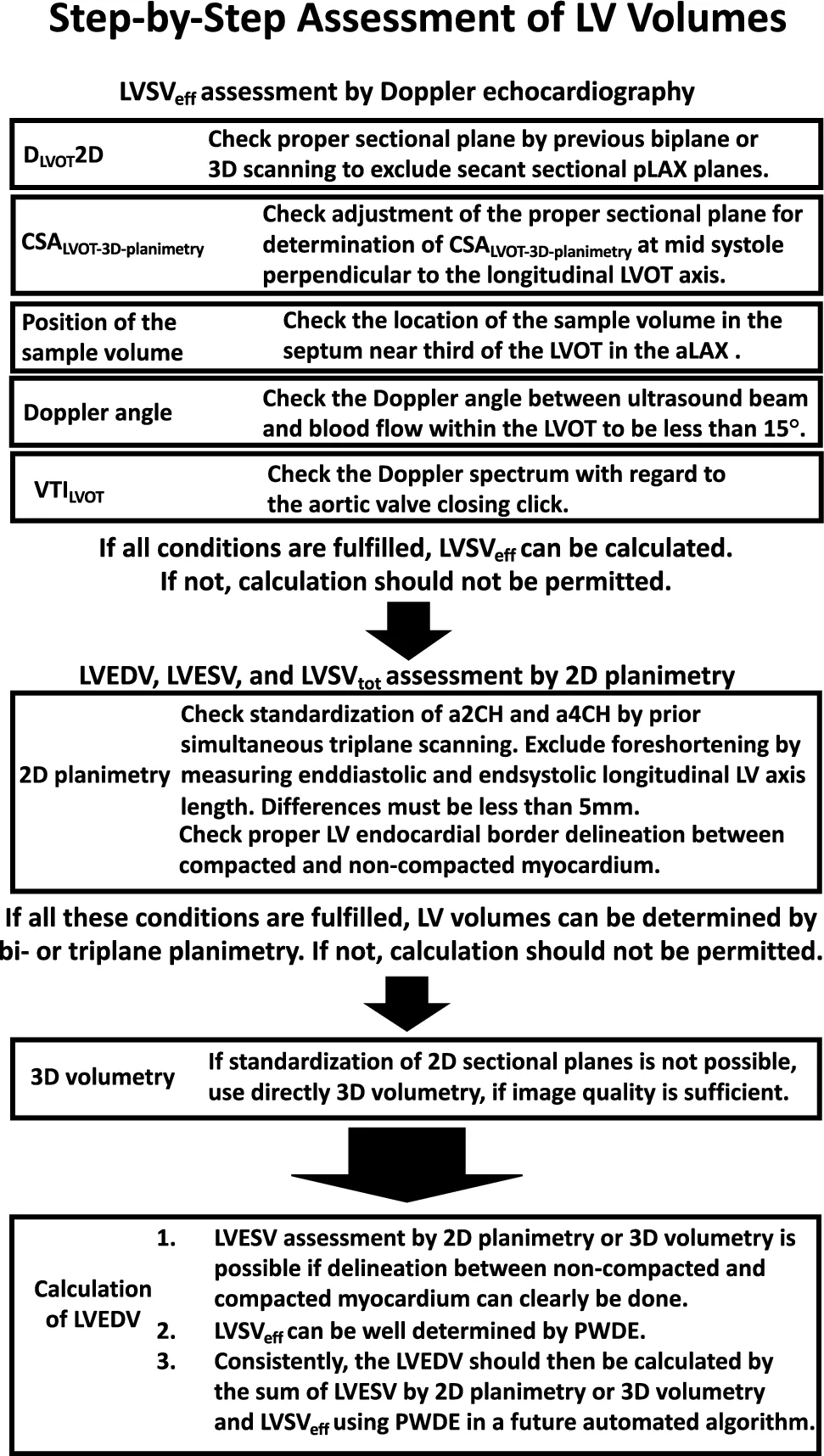
Proposal of a step-by-step algorithm for quantitative assessment of left ventricular volumes by echocardiography—D_LVOT_2D—diameter of the left ventricular (LV) outflow tract (LVOT) determined by 2D echocardiography, *pLAX* parasternal long axis, *CSA*_*LVOT-3D-planimetry*_ cross-sectional area of the LVOT determined by 3D echocardiography, *aLAX* apical long axis view, *LVSV*_*eff*_ effective left ventricular (LV) stroke volume, *a2Ch* apical 2-chamber view, *a4Ch* apical 4-chamber view, *LVESV* left ventricular end-systolic volume, *LVEDV* left ventricular end-diastolic volume, *PWDE* pulsed wave Doppler echocardiography

The robustness of LVSV measurement by Doppler echocardiography has already been reported in previous studies [19, 20, 40, 41]. In contrast to Doppler echocardiography, LVSV assessment by 2D and 3D echocardiography requires adequate delineation of the endocardial border during end diastole and end systole, clearly distinguishing between non-compacted and compacted myocardium. Poor delineation of the endocardial border is mainly due to the integration of the non-compacted myocardium into the myocardial wall during end diastole, resulting in an underestimation of LVEDV as well as LVSV [9–12]. In contrast, delineation of the endocardial border appears to be easier during end systole when there is complete compression of the intermyocardial spaces, representing only compact myocardium. Therefore, from a practical point of view considering these methodological limitations, a possible proposal for the assessment of LV volumes is shown in Fig. 4.

### Limitations

The present study included only echocardiographic data sets of healthy male athletes with sufficient image quality to allow an assessment of LV volumes by all echocardiographic modalities. Mean values of the present cohort will obviously differ from those of male non-athletes, women and patients with structural heart diseases. In addition, the number of athletes who have undergone cMRI is small which may affect statistics.

## Conclusion

The choice of echocardiographic method has a significant impact on LVSV. In general, the assessment of LVSV should be checked for plausibility. Further, the same echocardiographic method should be used to assess LVSV at follow-ups to ensure good comparability. Biplane LV planimetry by TTE in comparison to cMRI is a similar robust diagnostic tool.

## Data Availability

All data supporting the findings of this study are available within the paper.
